# Supplementary material for: Continentalic Acid Rather Than Kaurenoic Acid Is Responsible for the Anti-Arthritic Activity of Manchurian Spikenard In Vitro and In Vivo
Source: Int J Mol Sci. 2019 Nov 4;20(21):5488. doi: 10.3390/ijms20215488 (PMC6861925; doi:10.3390/ijms20215488)
Supplement: Supplementary file 1 [file ijms-20-05488-s001.pdf]

Continentalic Acid Rather Than Kaurenoic Acid Is Responsible for the Anti-Arthritic Activity of Manchurian Spikenard In Vitro and In Vivo

Riwon Hong [1,†], Kyoung Soo Kim [2,†], Gwang Muk Choi [3], Mijung Yeom [1], Bombi Lee [1], Sanghyun Lee [4], Ki Sung Kang [5], Hyang Sook Lee [1], Hi-Joon Park [1] and Dae–Hyun Hahm [1,6,7,*]

[1] Acupuncture and Meridian Science Research Center, Kyung Hee University, 02447 Seoul, Korea; smsmjhjh@naver.com (R.H.); myeom@khu.ac.kr (M.Y.); bombi@khu.ac.kr (B.L.); erc633@khu.ac.kr (H.S.L.); acufind@khu.ac.kr (H.-J.P.)

[2] Department of Clinical Pharmacology and Therapeutics, College of Medicine, Kyung Hee University, 02447 Seoul, Korea; labrea46@naver.com (K.S.K.)

[3] Department of Biomedical Sciences, Graduate School, Kyung Hee University, 02447 Seoul, Korea; alsdud335@naver.com (G.M.C.)

[4] Department of Plant Science and Technology, Chung–Ang University, 17546 Anseong, Korea; slee@cau.ac.kr (S.L.)

[5] College of Korean Medicine, Gachon University, 13120 Seongnam, Korea; kkang@gachon.ac.kr (K.S.K.)

[6] Department of Physiology, College of Medicine, Kyung Hee University, 02447 Seoul, Korea; dhhahm@khu.ac.kr (D.-H.H.)

[7] BioNanocomposite Research Center, Kyung Hee University, Seoul 02447, Korea

* Correspondence: dhhahm@khu.ac.kr; Tel.: +82-2-961-0366

† These authors contributed equally to this work.

Materials and Methods

1. HPLC Analysis.

A Waters Breeze system (Waters Co., Milford, MA, USA) equipped with a Waters 1525 binary HPLC pump and a Waters 2489 UV detector were used for HPLC analysis. The HPLC-grade reagents used were water containing 1% acetic acid and acetonitrile. All other reagents were of analytical grade. Quantitative analysis of the 50% ethanolic extract of the spikenard was performed using an isocratic reverse phase system. An INNO C18 column (4.6 × 250 mm, 5 μm) was used, and the column temperature was maintained at 30°C. The mobile phase was an isocratic elution with acetonitrile (solvent A) and water containing 1% acetic acid (solvent B) in a solvent ratio of 10:90 (v/v) for 40 min. A 10-μL aliquot was eluted, the UV detector was set at 205 nm, and

the flow rate was 1 mL/min. The HPLC chromatogram of the 50% ethanolic extract is shown in Figure S6. The content levels of continentalic acid and kaurenoic acid in the 0, 30, 50, 70 and 100% ethanolic extracts were indicated in Table S1.

2. Cell Viability Assay

Cell viability was determined using the EZ–Cytox® cell viability assay kit including water-soluble tetrazolium salt (WST)-1 (DaeilLab Service Co., Seoul, Korea). Briefly, human OA chondrocytes (or mouse RAW264.7 macrophages) were cultured overnight at a density of 6×10^3 cells per well in 96-well plates with low serum (1% FBS), followed by treatment with various concentrations of the extract up to 1000 μg/ml, and continentalic acid or kaurenoic acid up to 100 μM. After 24 h of incubation, 10 μl of WST-1 was added to each well. After a 1-h incubation at room temperature, the plates were read at 450 nm using a microplate reader (Molecular Devices Co., Sunnyvale, CA, USA). The results are expressed as a percentage of the WST-untreated control.

3. NO Assay

Nitric oxide (NO) formation in chondrocytes was determined by measuring nitrite (NO_2^-), a stable breakdown product of NO, using Griess Reagent System (Promega Co., Madison, WI, USA) according to the manufacturer's protocol.

Supplementary Figures and Table

Figure S1

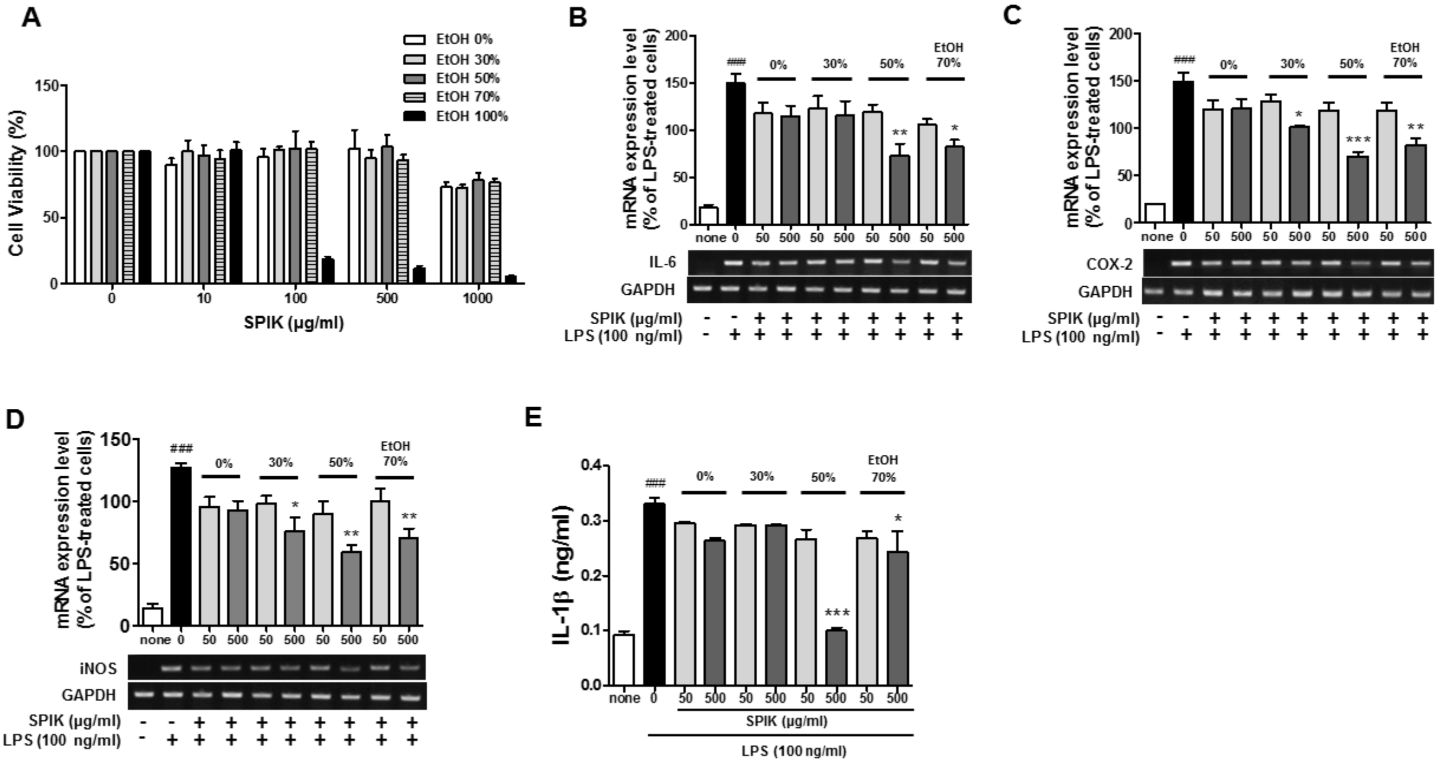

Figure S1. Effect of the ethanol content (0, 30, 50, 70 and 100 %) of Manchurian spikenard extraction solvent on the cell viability (**A**), the mRNA expression levels of IL-6 (**B**), COX-2 (**C**) and iNOS (**D**), and the secretion of IL-1β (**E**) protein in LPS-stimulated RAW294.7 cells. IL, interleukine; COX, cyclooxygenase; LPS, lipopolysacharride; iNOS, inducible nitric oxide synthase. $^{###}$ $p < 0.001$ vs. non-treated naïve cells (none); * $p < 0.01$, ** $p < 0.005$ and *** $p < 0.001$ vs. LPS-treated groups without treatments.

Figure S2

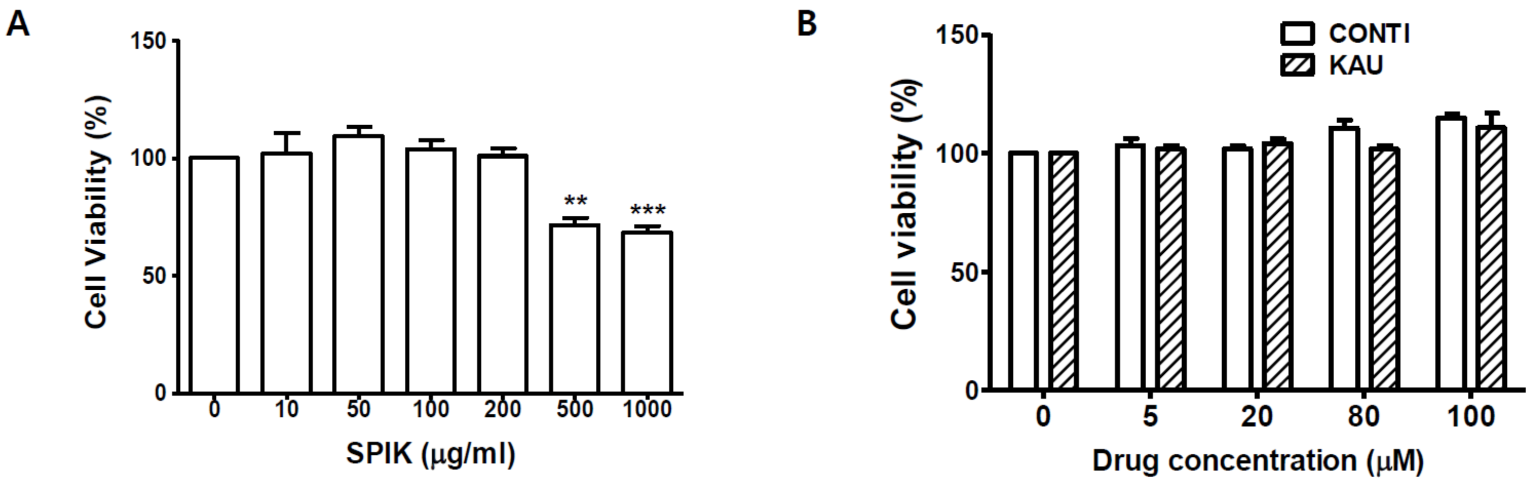

Figure S2. Effect of the 50% ethanolic extract of Manchurian spikenard (**A**), and continentalic and kaurenoic acids (**B**) on the human chondrocyte cell viability. SPIK, ethanolic extract of Manchurian spikenard; CONTI, continentalic acid; KAU, kaurenoic acid. Data are presented as the mean±SEM from at least three independent experiments. ** $p < 0.005$ and *** $p < 0.001$ vs. non-treated naïve cells

Figure S3

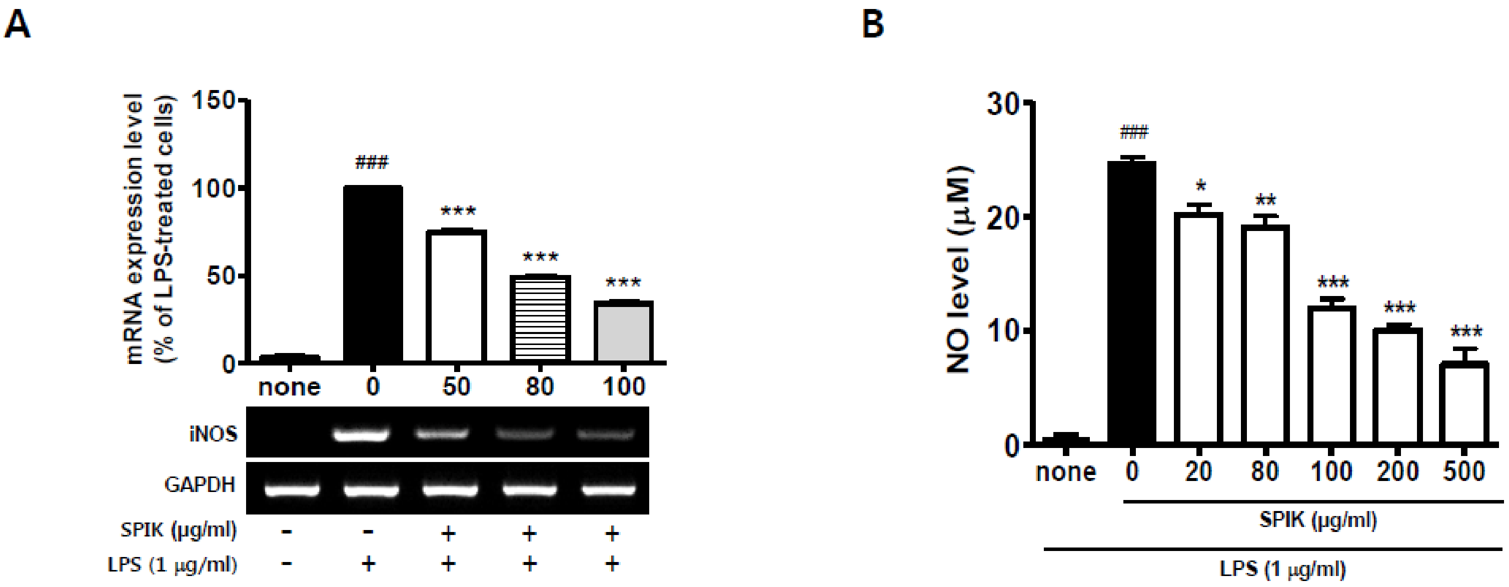

Figure S3. Effect of the 50% ethanolic extract of Manchurian spikenard on the mRNA expression levels of iNOS (**A**) and NO production (**B**) in LPS-stimulated RAW294.7 cells. SPIK: ethanolic extract of Manchurian spikenard; LPS, lipopolysaccharide; GAPDH, glyceraldehyde-3-phosphate dehydrogenase; iNOS, inducible nitic oxide synthase; NO, nitric oxide. $^{\#\#\#}$ $p < 0.001$ vs. non-treated naïve cells (none); * $p < 0.01$, ** $p < 0.005$ and *** $p < 0.001$ vs. LPS-treated group without treatments (0)

Figure S4

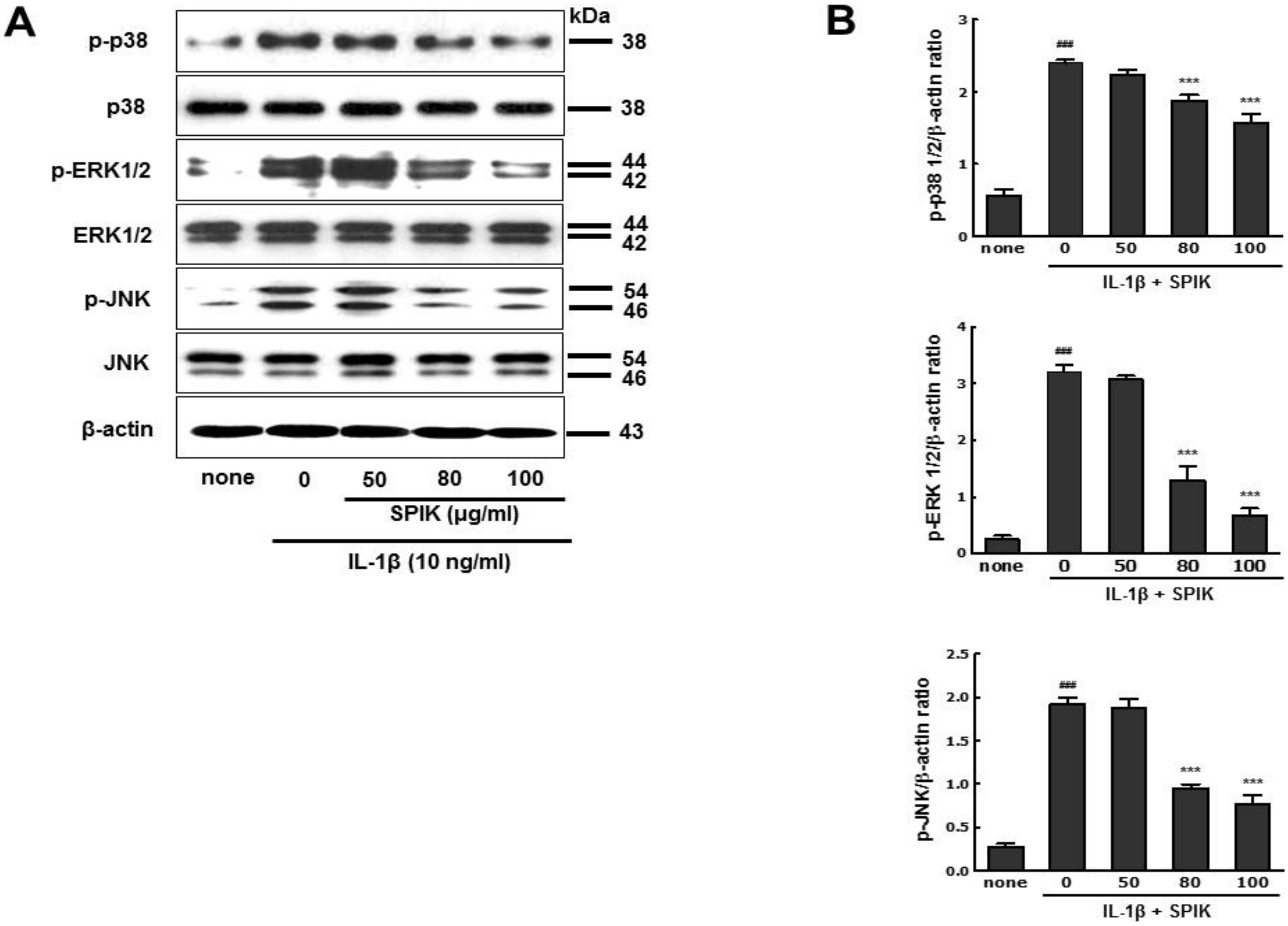

Figure S4. Western blot images (A) of *p*-p38, *p*-ERK and *p*-JNK MAP kinases, and their bar graphs (B) in IL-1β-stimulated human OA chondrocytes with the treatments of Manchurian spikenard extract. SPIK, ethanolic extract of Manchurian spikenard; ERK, extracellular signal-regulated kinase; p-ERK, phosphorylated ERK; JNK, jun N-terminal kinase. $^{###}$ $p < 0.001$ vs. non-treated naïve cells (none); *** $p < 0.001$ vs. IL-1β-treated group without treatments (0).

Figure S5

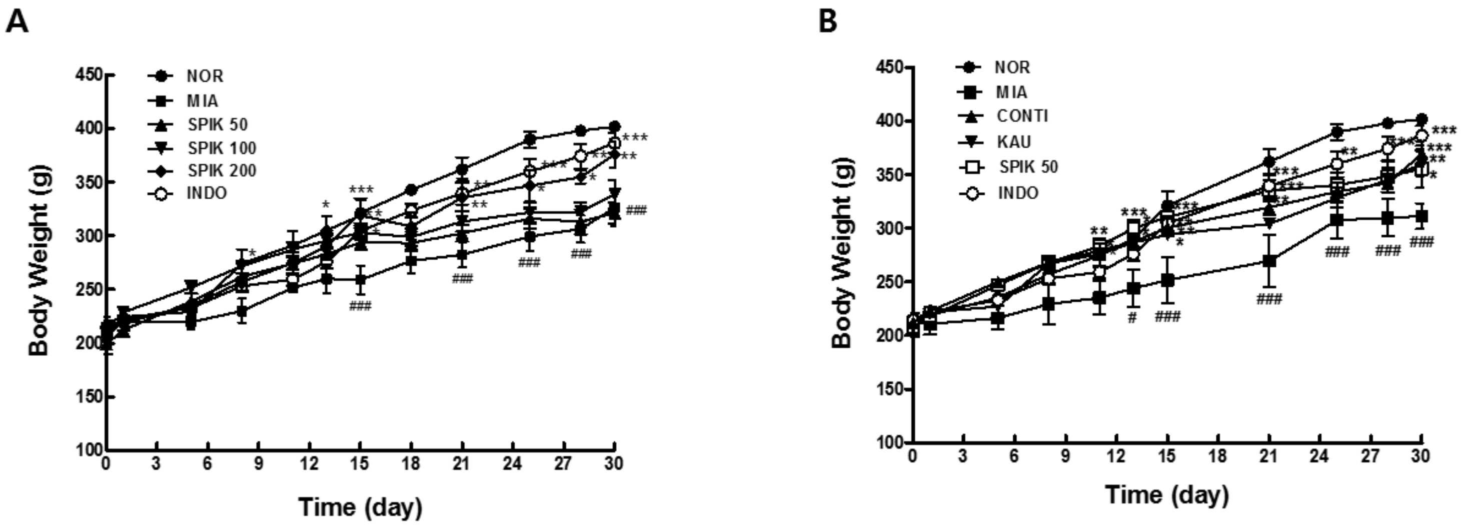

Figure S5. Effects of 50% ethanolic extract of Manchurian spikenard (A), and continantalic and kaurenoic acids (B) on body weights monoiodoacetate (MIA)-induced osteoarthritic rats. Different doses of the spikenard extracts were treated by oral administration in A and i.p. injection in B. SPIK, ethanolic extract of Manchurian spikenard; CONTI, continentalic acid; KAU, kaurenoic acid; INDO, indomethacin. $^{###}$ $p < 0.001$ vs. non-treated normal group (NOR); * $p < 0.05$, ** $p < 0.01$, *** $p < 0.001$ vs. vehicle-treated MIA group without SPIK, CONTI or KAU treatment.

Figure S6

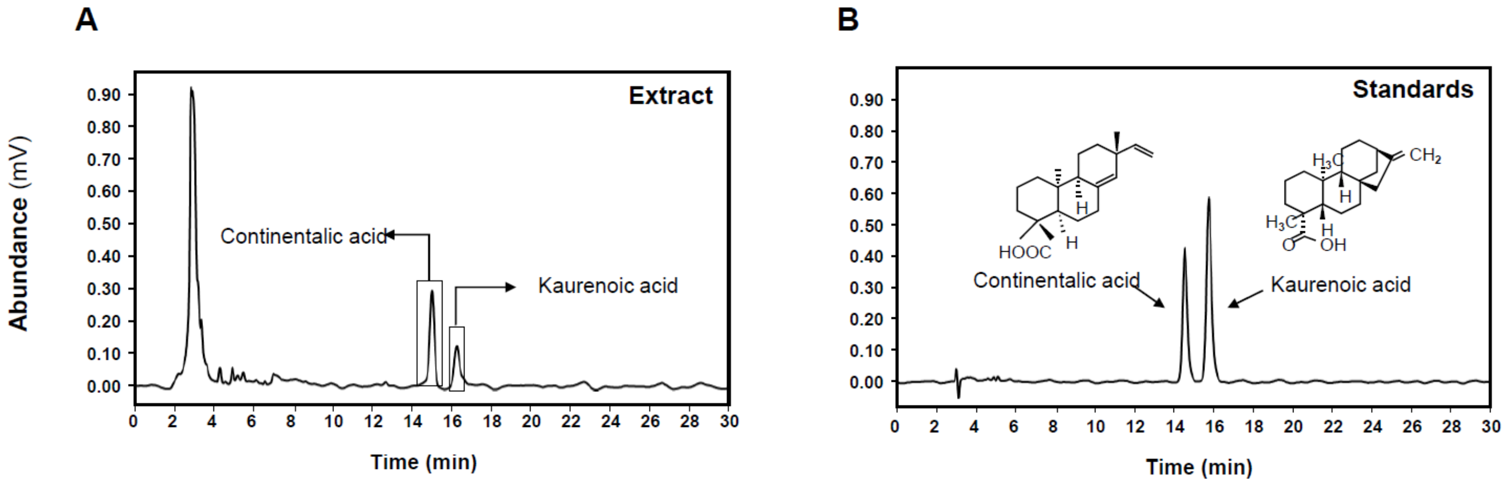

Figure S6. HPLC chromatograms of the 50% ethanolic extract (**A**) of Manchurian spikenard and its reference compounds, continentalic acid and kaurenoic acid (**B**).

Figure S7

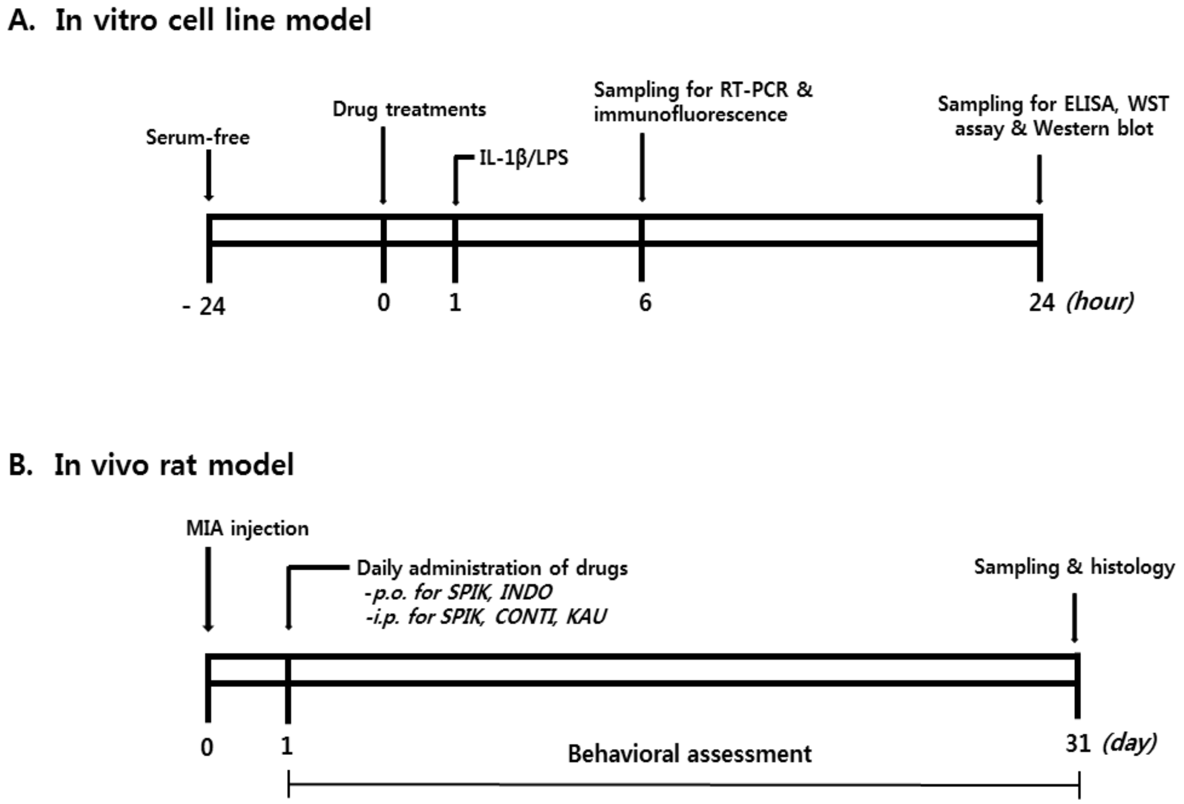

Figure S7. Experimental schedules of an in vitro model (**A**) of cartilage degradation using IL-1β-stimulated human chondrocytes, and an in vivo rat model (**B**) for the study of human osteoarthritis. Knee arthritis was developed by intra-articular injection of monoiodoacetate (MIA), which induced chondrocyte death in the articular cartilage and thus led to develop functional joint impairment in the rats. LPS, lipopolysacharride; ELISA, enzyme-linked immunosorbent assay; WST, water-soluble tetrazolium salt; CONTI, continentalic acid; KAU, kaurenoic acid; SPIK, ethanolic extract of Manchurian spikenard; INDO, indomethacin.

Table 1. The contents of continentalic acid and kaurenoic acid in the 0, 30, 50, 70 and 100% ethanolic extracts of Manchurian spikenard (*Aralia continentalis* Kitag.)

EtOH% in extraction solvent	Continentalic Acid (mg/g-ext)	Kaurenoic Acid (mg/g-ext)
0	1.289 ± 0.133	0.151 ± 0.044

30	5.494 ± 0.543	2.258 ± 0.224
50	19.041 ± 0.251	6.726 ± 0.345
70	13.798 ± 0.217	5.369 ± 0.112
100	12.097 ± 0.200	3.378 ± 0.253

EtOH, ethanol; ext, extract
